# Preprints as a driver of open science: Opportunities for Southeast Asia

**DOI:** 10.3389/frma.2022.992942

**Published:** 2022-09-26

**Authors:** Dasapta Erwin Irawan, Hilyatuz Zahroh, Iratxe Puebla

**Affiliations:** ^1^Institut Teknologi Bandung and RINarxiv, Bandung, Indonesia; ^2^Universitas Yarsi and RINarxiv, Central Jakarta, Indonesia; ^3^ASAPbio, Cambridge, United Kingdom

**Keywords:** research integrity, open science, preprints, reproducibility, Southeast Asia, open access, diversity

## Abstract

Southeast Asia is an emerging force of open access scholarly output. For example, Indonesia is in a tight competition with United Kingdom as the largest publisher of open access journals and the second largest producer of open access articles in the world (according to DOAJ and the COKI OA Dashboard, respectively). However, this support for open practices is not yet reflected in institutional research policies in Southeast Asian countries, which still rely on criteria influenced by world university rankings that focus on publication outputs and do not incorporate elements related to research culture, integrity, or open science. Preprints have gained increasing attention across disciplines in the last few years, but they are still not included in institutional policies in SouthEast Asia. This paper discusses the potential for preprints to be a driving force for open science and for quality and integrity in scholarly outputs from Southeast Asia. There is a fledgling preprinting culture in the region, catalyzed by the RINarxiv preprint server in Indonesia and the Malaysia Open Science Platform. We argue that preprints have many advantages: opportunities for open access and for researchers to maintain copyright to their work, wide dissemination, encouraging feedback and critical thinking, and community governance. With these advantages, preprints can become a fast and open communication hub between researchers and all stakeholders in the research process. We recommend regulatory and practical steps to incorporate preprints into science policy and researchers' practices as an effort to promote research integrity, open data and reproducibility.

## Introduction

Over the last two decades, a major theme for universities in Southeast Asia has centered on strengthening their reputation and global standing (Asian Development Bank, 2011). To achieve this goal, there is a recognition that all stakeholders should put more focus on research integrity, transparency and accountability (Stagars, 2016; UNESCO, 2022). However, the recognition for those three components comes in contrast with current practice in most universities in the region, which focus their attention on World University Rankings (WUR) as their indicator of quality and not on research integrity practices (Irawan and Abraham, 2021). In addition, Southeast Asia research ecosystems have encountered common challenges related to limited research funding coming mainly from the government, many potential students coming from middle to low-income backgrounds, and a considerable proportion of researchers trained at small to mid-size universities with various quality of educational backgrounds leading to disparities in implementation of best research practices.

There is a recognition that research integrity and quality are supported by transparency in the research process, and over the last few years there has been increased awareness and adoption of open science practices worldwide (Armeni et al., 2021; Robson et al., 2021). There has also been growing attention to open science in the Southeast Asian region. These efforts have been driven by research communities from various countries, such as the South East Asia Network of Open Science (SEANOS), the Indonesia Open Science Team, and the Malaysian Open Science Platform (MOSP) (Onie, 2020). These organizations promote transparency and accountability in research as a means of driving reputation and integrity. However, the values of transparency and accountability are still rarely considered or explicitly measured as part of research assessment in the region, which is instead very much driven by practices and metrics aiming to achieve a higher position in world rankings such as Quacquarelli Symonds (QS), Times Higher Education World University Ranking (THES), and the Academic Ranking of World Universities (ARWU).

But recognition for open science and its importance for research and society has continued to grow. In 2021, UNESCO released its recommendations on open science (UNESCO, 2021). The document presents four values: quality and integrity, collective benefit, equity and fairness, diversity, and inclusiveness. In this opinion piece, we argue that preprints constitute an important tool within open science that fits all the values outlined in the UNESCO recommendations, and thus can be used strategically to promote the reputation and visibility of research from Southeast Asia.

## Preprints: A growing presence globally and in Southeast Asia

While physicists have used the preprint server arXiv for decades to share their latest papers, the use of preprints in other disciplines was marginal until recently. Things started to change over the last decade: the preprint server for biology bioRxiv launched in 2013 and attracted an increasing number of papers year-on-year. In parallel to bioRxiv's growth, dozens of new preprint servers launched 2016 onwards, including a number of platforms supported by the Center for Open Science (COS) (Nosek, 2022). This rapid expansion has been described as the “second wave of preprints” (Scholarly Kitchen, 2020), characterized by an increasing popularity of preprints among researchers, but also heightened interest by publishers, which launched their own preprint servers, e.g., preprints.org by MDPI (MDPI, 2016), or acquired existing servers, e.g., Elsevier acquired SSRN (Elsevier, 2022a). In parallel to this, different groups developed their own community-driven preprint servers, some of them with a focus on providing visibility to research from specific geographical regions, such as IndiaRxiv in India and INArxiv in Indonesia. Currently we have seven regional scope preprint servers (Table 1).

**Table 1:** Regional scope preprint servers.

**No**	**Preprint servers**	**Link**	**Regional**
1	RINarxiv	https://rinarxiv.lipi.go.id	Indonesia and SE Asia
2	Arabixiv	https://arabixiv.org/	Arab region/peninsula
3	ChinaXiv	http://chinaxiv.org/home.htm?locale=en	China
4	Scielo Preprints	https://preprints.scielo.org/index.php/scielo	Latin America
5	AfricaRxiv	https://info.africarxiv.org/	Africa continent
6	Jxiv	https://jxiv.jst.go.jp/index.php/jxiv	Japan
7	IndiaRxiv	https://ops.iihr.res.in/index.php/IndiaRxiv/index	India and South Asia

**Figure 1:**
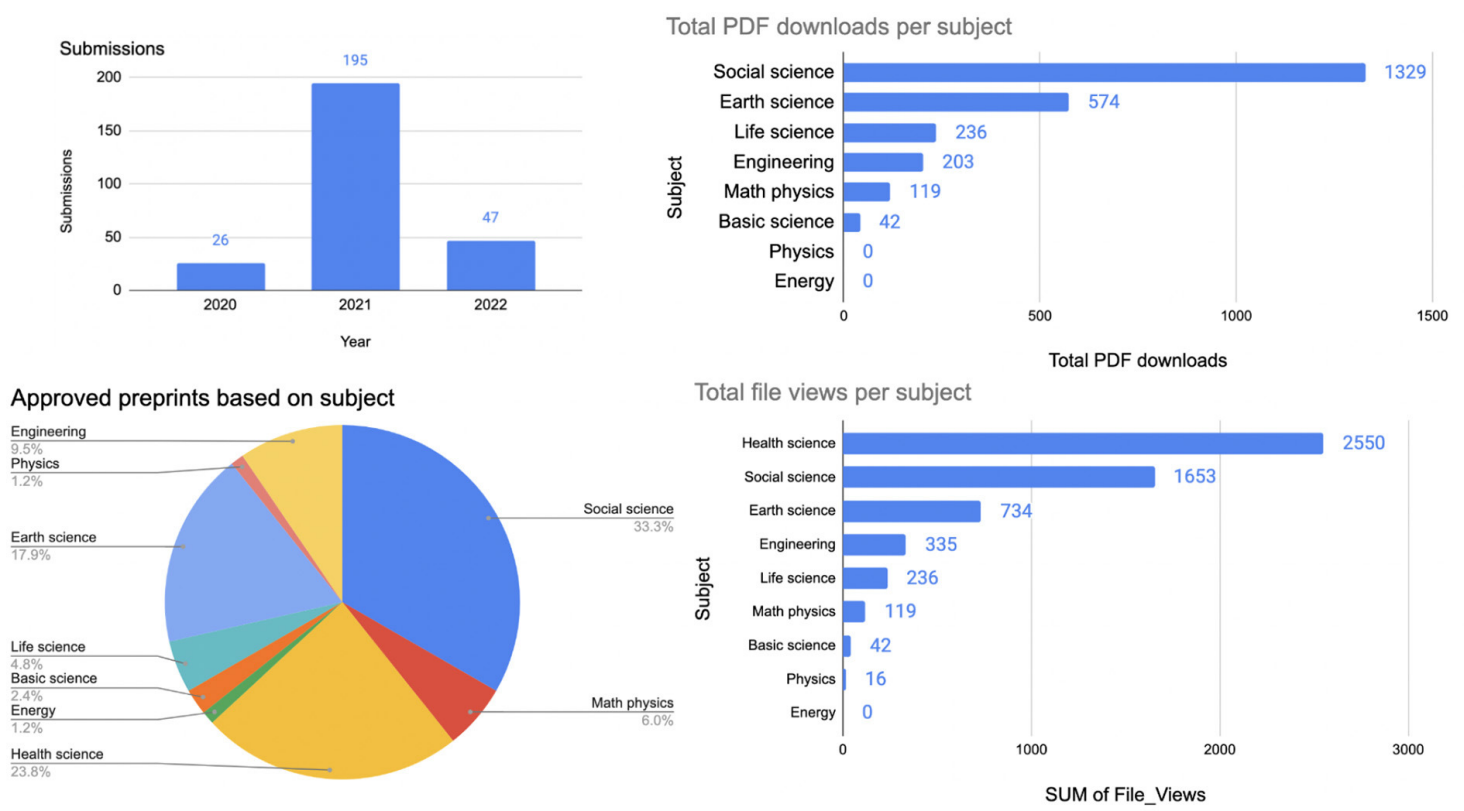
RINArxiv infographic from May 21, 2020 until June 30, 2022.

INArxiv was launched on August 17, 2017 as a platform to foster interaction between Indonesian open science activists and the international community. The server was launched on the open-source Open Science Framework infrastructure by the Center for Open Science (Science C for O, 2022). The visibility of the server grew supported by community outreach around open science and in the following 2 years, INArxiv received more than 1,000 documents. At the end of 2019, COS asked each of the servers it hosted to raise funds to support maintenance costs for the platforms, at a level proportional to the number of submissions (Mallapaty, 2020). This imposed a heavy burden for INArxiv which at the time operated as a fully community-run initiative, and in January 2020, INArxiv officially stopped operating. The community behind the server was keen to provide a platform for researchers in the region to interact and share their work, and it pursued conversations with several stakeholders in Indonesia. These contacts led to support from the government agency Pusat Dokumentasi dan Informasi Ilmiah (PDDI), a division of Indonesian Institute of Sciences/LIPI, now renamed as National Research and Innovation Agency/BRIN (BRIN, 2022), which agreed to help with server hosting, and on May 21, 2020 INArxiv was reborn under the name RINarxiv. The name “RIN” refers to the Repositori Ilmiah Nasional (the National Scientific Repository) which is also hosted by PDDI BRIN. In its 2 years of operation, RINarxiv has hosted 84 preprints by Indonesian authors, and attracted over 5,600 file views and close to 5,000 PDF downloads (Figure 1).

## Driving open science through preprints

There are many reasons why preprints are a powerful tool to drive open science practices. Preprints allow work to be shared with the community as and when it is ready, allowing faster dissemination and in turn supporting scientific progress. Preprints provide researchers with more control and freedom about the communication of their research compared to journal publication, including:

- ***What*** work to share: preprint servers do not evaluate interest level or novelty so preprints can report many different research outputs, including early or ongoing work or negative results that may be tricky to publish in a journal.
- ***When*** to disseminate research findings: preprint servers usually post preprints within a few days, a time frame much shorter than that of a journal's editorialprocess.
- ***Which format*** to use: preprint servers do not impose specific format or length restrictions as it's often the case at journals.

### Preprints broaden access

Preprints are free to post and free to access. They are free of the pay walls or subscriptions that often apply to journal articles, and thus, allow researchers to make their work accessible to readers all over the world. In the case of many journals, the publisher will require that the authors transfer their rights over the work to the publisher, meaning that once the article is published, the researcher may be limited in how they can use the article they have themselves conceptualized and written. Preprints do not require a copyright-transfer agreement and thus, the author retains full rights to their preprint. Preprint servers offer authors either a variety of license options (e.g., RINarxiv, bioRxiv or medRxiv), or require a creative Commons CC BY license [e.g., Research Square (2022)] where the authors retain rights to the work but allow others to reuse the paper provided attribution is given to the authors (Asapbio, 2022a,b).

Indonesia has embraced open access as a publication model and is one of the largest producers of open-access articles (COKI, 2022; DOAJ, 2022). Preprint servers provide an additional channel to make research outputs open access, either prior to journal publication, or to self-archive copies of manuscripts accepted at a journal and make that freely available (the green Open Access route) even if the journal article is behind a paywall.

### Preprints support reproducibility

The UNESCO open science recommendations highlight preprints as a tool to improve quality and reproducibility across different stages of the research process. Through preprints, researchers can share their work well in advance of journal publication, opening opportunities for community feedback while the research is still ongoing. By virtue of their free availability, preprints allow researchers from a wide range of backgrounds and communities to react to the work, suggest improvements or point out oversights. Because the feedback can be incorporated before the work is even submitted to a journal, preprints can strengthen the research at an earlier stage and lead to greater integrity and quality in the eventual journal publication for that work.

While it can be challenging to publish null and negative results in a journal, preprint servers allow researchers to easily share unexpected observations or negative or inconclusive results. The preprint can ensure that those data are available to the research community, which supports reproducibility and reduces publication bias.

### Preprints are an open-science tool for everyone

Researchers have mostly adopted preprints to share their work faster and more openly than was possible through journal publication, but preprints hold much more promise to materialize the benefits of open science practices for everyone involved in the research process. Preprints provide a lever to progress toward the goals outlined in the UNESCO recommendations (UNESCO, 2021) as they create a channel for science communication accessible to all stakeholders, encouraging collaboration and feedback, and linking the different outputs generated at each stage of a research project (Table 2).

**Table 2:** Benefits of preprints to the different stakeholders involved in research.

**Stakeholders**	**UNESCO open science recommendations**						
	1 Promote a common understanding of open science, benefits & challenges, diverse paths to open science	2 Develop an enabling policy environment for open science	3 Invest in open science infrastructures & services	4 Invest in human resources, training, education, digital literacy & capacity building for open science	5 Foster a culture of open science & align incentives for open science	6 Promote innovative approaches for open science at different stages of the scientific process	7 Promote international & multi-stakeholder cooperation in open science
Funders	Preprints are an immediate proof of productivity, allowing the evaluation of the most immediate research outputs at the end of the grant period.					
	Preprint are free and provide a low-cost open access option; a wider range of outputs can be shared, bringing a higher return on investment for grants.					
	Preprints support FAIR dissemination, they make the latest research results widely findable and accessible, reducing the risk of duplication or unnecessary repetition.					
Research Institutions	Preprints provide wide reach and increased transparency to the dissemination of the latest research findings, which can drive institutional reputation.					
	Preprint servers align to many of the goals and purposes of institutional repositories.					
	Preprints enable interactions across stakeholders earlier in the research process.					
Academic Journals	Editorial policies that accept preprints support wide & early sharing of research outputs.			Opportunities to innovate *via* transfers from preprint servers to journals, or invitations to submit preprints from emerging fields.		
				Preprints provide early exposure to the work and drive early citations when the journal article appears.			
Research Community	Preprints provide researchers new ways and freedom to share their work. Preprints set the priority of claim to prevent scooping.				Preprints enable early interactions & collaborations among researchers.		
					The assessment is focused on the scientific content and free of journal-title proxies, which helps develop critical thinking and review skills.		
					Preprint offers solution for researchers with limited APC funding. They can publish the manuscript in a non-OA journal and upload the preprints or author's accepted version to preprint server as the OA version.		
Society	Preprints allow broad dissemination *via* messaging groups (e.g., Whatsapp, Telegram) or social media (e.g., Twitter, Facebook).			Non-specialized readers, such as the public or journalists, can comment or engage with the preprint directly.			Preprints remove barriers for citizen science groups to share their work.

For funders, preprints provide early proof of productivity and a higher return-on-investment as they allow the sharing of outputs that have traditionally not made it into journal publication. By allowing the inclusion of preprints, funders can access the researcher's most recent accomplishments, focusing their assessment on the science and on the latest developments in the field, rather than on a particular target journal publication that may report work completed months if not years prior. With regard to institutions, support for preprints signals support for avenues for collaboration and feedback among researchers. In addition, considering preprints as part of hiring processes provides institutions with an opportunity to evaluate the applicant for their most recent and relevant research, allowing better informed decisions on their suitability for the particular role.

Preprints can also benefit journals. By partnering with preprint servers, journals support open science practices and can innovate in their processes. Many journals have implemented workflows to transfer manuscripts from preprint servers, as is already in place for bioRxiv and medRxiv (Sever et al., 2019). Such partnerships bring opportunities to the journals to widen their reach into new areas of research, for example by inviting the submission of preprints in up-and-coming new fields (Singh-Shepherd, 2022).

Open science aims to also open both access and opportunities to contribute to scientific knowledge for societal actors. Preprints drive this goal as they are freely available not only to the research community but also to non-governmental organizations, the media, and the broader public. This transparency supports trust in research as a whole. Preprints open up the iterative process of science, where different results, revisions, interactions and even corrections are required before a finding can be established as scientific evidence; this can help the public learn more about how the scientific process works. Through preprints, citizen science groups (which often carry out important data-collection work in fields such as environmental sciences), no longer face financial hurdles to access the latest findings or to share their own data widely so that they can inform further research or even policy.

## Making the benefits of preprints a reality for open science in Southeast Asia

The experience from RINarxiv in Indonesia suggests that the use of preprints in the region is currently confined to those researchers and communities exposed to open science practices, for example, those involved with the Indonesian Open Science Community. There is thus much work left to do to raise awareness of preprints within local research communities.

**Figure 2:**
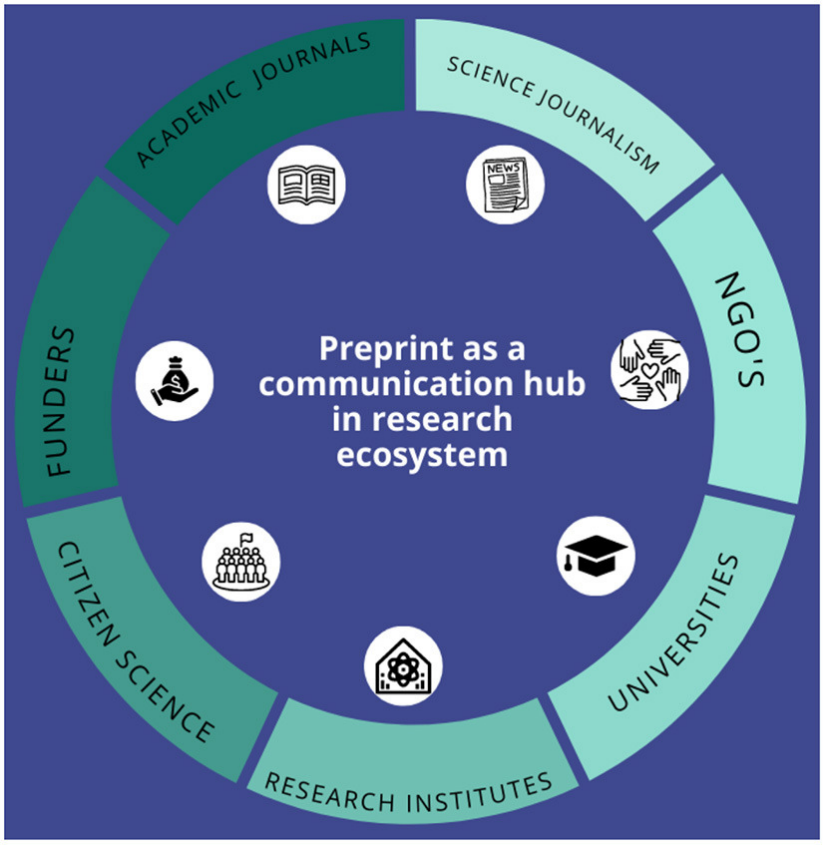
Preprints as a central communication hub in the research ecosystem.

Our vision for preprints in Southeast Asia is that they will bring more speed, freedom, and reach to science dissemination by becoming the central communication hub for all stakeholders in the research ecosystem (Figure 2). In order to ensure that preprints fulfill this promise, it will be important for all stakeholders in the region to signal support for this open science practice. We outline below specific steps that each stakeholder can take in support of preprints (Table 2).

### Funders

Funder policies that recognize preprints have been influential in North America and Europe (Matthews, 2018; Kaiser, 2022), and similar steps by funding agencies in Southeast Asia would likely bring increased use of preprints by researchers in the region. The Indonesian Ministry of Education, Culture, Research, and Technology, a major funder of research in the country, requires that grantees submit copies of their publications by the end of the grant period. An expansion of this policy to allow the inclusion of preprints would allow researchers to submit their latest work independent of its status within the journal's editorial process; this would be particularly beneficial for early career researchers who are still building their publication portfolio and for whom a delay in a journal's process can be particularly detrimental. Such a policy would also signal the ministry's commitment to open science practices by recognizing copies of research papers shared in a freely-available format. In addition, researchers can enrich the preprint with data, code, and methods (e.g., in the form of a laboratory notebook) as supplementary materials at no additional cost, providing a richer overview of their productivity compared to the format restrictions that may apply to journal publications.

### Institutions

Many institutions have publication requirements for graduation within a PhD program. This means that graduation may be delayed or jeopardized if the publication of the doctoral work is delayed during the journal's process. We recommend that institutions in SouthEast Asia accept preprints as part of PhD graduation requirements, allowing students to show proof of their research productivity as soon as they are ready to disseminate their work to the community. A number of doctoral schools in France recognize preprints recommended by the preprint review community Peer Community In as of the same value as good-quality journal publications (Asapbio, 2022a,b), and similar policies at universities in Southeast Asia would provide a strong support for preprints and open science.

In addition, universities in Southeast Asia could include preprints in tenure and promotion processes for their faculty members, as it is already in place in a number of institutions (Asapbio, 2022a,b).

Institutions can also support awareness and understanding of preprints by hosting events that discuss preprints or collaborating with local open science groups such as the SouthEast Asia Network for Open Science (SEANOS) and preprint servers like RINarxiv to run informational sessions and workshops.

Box 1 **Opportunities for early career researchers to raise their profile *via* preprints.**

Indonesian researcher Putu Sukma Kurniawan shared his work on indicators for sustainability accounting in small and medium enterprises as a preprint on INArxiv (Kurniawan, 2019) on March 2019. Dr Kurniawan shared his preprint widely on social media and also regularly develops videos related to his work, which he shares on Youtube (Kurniawan, 2022a). This broad activity disseminating research resulted in an invitation from the online media platform The Conversation to participate as a contributor (Kurniawan, 2022b). Kurniawan also supports outreach about preprints and has shared his experience in events such as the Preprint month activities hosted by RINarxiv during the summer of 2021.

### Principal investigators

Principal investigators can incorporate preprints into the communication plans for their group's research and post their latest papers on a preprint server. When considering where to post the preprint, researchers may consider whether the work has a particularly strong regional relevance, in which case posting it at a server with a regional focus such as RINarxiv can ensure wider visibility with the intended audience.

Team leaders can also encourage group members to engage with preprints by bringing papers posted at preprint servers for discussion at lab meetings or journal clubs.

When hiring for new roles in the group, team leaders can clearly invite applicants to include preprints in their applications, as has already been done in job ads for a number of positions (Asapbio, 2022a,b). While peer-reviewed journal publications will still be a significant factor in the selection process, allowing preprints in the applicant's CV allows them to show their latest research contributions, even if they are still undergoing peer review or are not yet submitted to a journal.

### Early career researchers

If their supervisor and co-authors are on board, early career researchers can post their paper as a preprint and share it widely to encourage feedback. By taking proactive and innovative steps to promote their preprint, researchers can raise their profile and the visibility of their work within the research community (Box 1).

Early career researchers may not be the ultimate decision makers in the decision to post a preprint, but they can influence this choice in a number of ways. We encourage early career researchers to initiate conversations with their supervisors about the use of preprints—-this preprinting guide developed by and for early-career researchers includes tips for how to start and handle these conversations (Ettinger et al., 2022).

Early career researchers can also join a preprint community or a preprint journal club to learn more about preprints, contribute to outreach, and develop critical thinking and reviewing skills, e.g., Peer Community In (2022) or Synbio (2022). These initiatives would also help preprint services like RINarxiv to engage with the community interested in contributing to screening of submissions and outreach activities.

### Journals

It is important for journals to have clear editorial policies indicating whether they accept preprints—examples of editorial policies that support preprints include those by the publishers Elsevier (2022b), Springer (2022), and Wiley (2022), which apply across their portfolios of journals.

Several journals from Southeast Asia have also developed policies which encourage the posting of preprints, such as the *Indonesian Journal of Health Administration* (JAKI, 2022) or the *Indonesian Journal of Architecture*- EMARA (2022). It will greatly support awareness of preprints among researchers in the region if other regional journals followed their lead to also develop public preprint policies. In addition, these journals can also engage with preprints by developing partnerships with servers with a regional focus like RINarxiv, to invite submissions to the journal or explore preprint server-to-journal transfer workflows. This type of partnership would strengthen collaboration across organizations in the region and provide those journals with opportunities to widen their audience, or even increase their pool of potential reviewers by tapping into those researchers who contribute reviews on preprints.

## Looking ahead

Preprints bring benefits to all stakeholders, but their future also depends on the engagement of all stakeholders.

We should ensure that the principles of diversity and equity anchor ongoing and future practices around preprints. Much can be learnt from the journal publication ecosystem, where important progress has been made in open access but where unintended consequences have also surfaced around inequity, for example, regarding publication fees that hinder who can publish open access in certain journals (Cole et al., 2022). However, for researchers with limited funding for APC, they can choose to publish in a no-APC open access journal or, if necessary, they can publish the paper in a non-open access journal and upload the manuscript to a preprint server and thus, provide readers with a copy of the manuscript in open access format via that server.

Preprints are a science communication tool that allows everyone to freely disseminate their work, but it will be important to ensure that the voices of communities from a broad spectrum of geographical regions are represented if preprints are to meet the specific needs of those diverse communities. As the UNESCO recommendations highlight, there should be diverse paths to open science, and practices that work for communities in some regions may not be best suited for others. Researchers and stakeholders in SouthEast Asia should work together to discuss ongoing efforts and challenges, and to proactively develop best practices for preprints that take their needs into consideration. Encouraging discussion across stakeholders at regional, national, and cross-national level *via* workshops and related forums will ensure a broad diversity of voices is represented and facilitate buy-in toward recognition of preprints.

Developing awareness and support for preprints is likely to require different approaches according to the current stage of open science in different regions. In countries such as Indonesia where there is strong grassroots support for open science, a bottom-up approach of outreach and advocacy may provide ways to encourage further discussion at institutional and national level. On the other hand, in countries with existing open science frameworks, such as Malaysia with its Open Science Alliance and the Malaysia Open Science Platform or MOSP (Akademi Sains Malaysia, 2022), incorporating preprints into those frameworks and leveraging this top-bottom approach may provide ways to drive adoption.

It will also be important to embed preprints with the existing training and workflows of researchers. If researchers view preprints as one more step to handle rather than a tool within their science communication workflow, it will be difficult to build engagement. Incorporating preprints in current training and education for researchers such as the Responsible Conduct of Research program introduced in Malaysia, would provide opportunities to place preprints as part of best research practices.

We should also nurture a culture of researcher responsibility and critical thinking. Opening up the research cycle and sharing drafts of manuscripts prior to journal publication does not mean that the quality of the research should be disregarded. Preprints, as any other research work, should be carefully prepared and written, with the same care as would be applied to a manuscript submitted to a journal. By sharing carefully prepared preprints, researchers can build up their reputation and attract engagement with their work from members of their community. Institutions can support researchers by educating students and faculty about preprints, the steps they should take before posting a preprint, and how they can use preprints as a tool to gain early feedback and increase the rigor of their research works.

Coaching also needs to be done to the managers of academic journals. It is important to emphasize to editors the concept of preprints as a form of communication of preliminary drafts and of self-archiving of accepted manuscripts - and not a research output that constitutes prior publication for the purpose of consideration at a journal. In this way, it is hoped that we can increase the number of preprint-friendly journals.

The values of transparency and rigor that preprints enable must be the main drivers for a broader preprint culture in Southeast Asia. It will be important to create an environment where those involved in the assessment of researchers (e.g., funders, institutions) clearly signal that they value open science practices when completing their evaluations, and that they recognize where preprints are used as a tool to increase research reproducibility and rigor. This involves allowing researchers to include preprints as proof of productivity, recognizing publications -including preprints—that report negative or confirmatory results and recognizing community engagement and collaborations enabled by preprints as valuable research contributions.

Indonesia has been noted as one of the leaders in the open-access movement (Cole et al., 2022). The country and others in the region can follow on those steps and make preprints a tool for transparency and reproducibility, providing a model for how open science practices can go hand-in-hand with integrity in research. Southeast Asia should seize the opportunity to leverage preprints to continue its leading role in adoption of open science practices.

## Author contributions

All authors listed have made a substantial, direct, and intellectual contribution to the work and approved it for publication.

## Funding

The publishing of this article was supported by funding from Institut Teknologi Bandung via PPMI 2022 scheme under Research and Community Outreach Fund 2022 (PPMI 2022) granted to Applied Geology Research Group, Faculty of Earth Sciences and Technology.

## Conflict of interest

DI and HZ are the moderators of RINarxiv. IP is an employee of ASAPbio, an organization that promotes the use of preprints in the life sciences.

## Publisher's note

All claims expressed in this article are solely those of the authors and do not necessarily represent those of their affiliated organizations, or those of the publisher, the editors and the reviewers. Any product that may be evaluated in this article, or claim that may be made by its manufacturer, is not guaranteed or endorsed by the publisher.

## References

[B1] Akademi Sains Malaysia, (2022). Malaysia Open Science Platform (MOSP) – Charting The Way Forward For Malaysia Open Science. Available online at: https://www.akademisains.gov.my/mosp

[B2] Armeni K. Brinkman L. Carlsson R. Eerland A. Fijten R. Fondberg R.. (2021). Towards wide-scale adoption of open science practices: The role of open science communities. Sci. Public Policy 48, 605–611. 10.1093/scipol/scab039

[B3] Asapbio A. (2022a). Preprint Licensing FAQ. ASAPbio. Available online at: https://asapbio.org/licensing-faq

[B4] Asapbio A. (2022b). University Policies and Statements on Hiring, Promotion, and Journal License Negotiation. ASAPbio. Available online at: https://asapbio.org/university-policies

[B5] Asian Development Bank (2011). Higher Education Across Asia: An Overview of Issues and Strategies. Available online at: https://www.adb.org/publications/higher-education-across-asia-overview-issues-and-strategiesstrategies

[B6] BRIN (2022). BRIN Official Web Page. Ristekdikti. Available online at: https://international.brin.go.id

[B7] COKI (2022). COKI Open Access Dashboard - COKI. COKI. Available online at: https://openknowledge.community/dashboards/coki-open-access-dashboard

[B8] Cole N. L. Reichmann S. Ross-Hellauer T. (2022). Global Thinking. ON-MERRIT recommendations for maximising equity in open and responsible research. Zenodo. 10.5281/zenodo.6276753

[B9] DOAJ (2022). Directory of Open Access Journals. Available online at: https://doaj.org

[B10] Elsevier (2022a). Elsevier Acquires the Social Science Research Network (SSRN), the Leading Social Science and Humanities Repository and Online Community. Available online at: https://www.elsevier.com/about/press-releases/corporate/elsevier-acquires-the-social-science-research-network-ssrn,-the-leading-social-science-and-humanities-repository-and-online-community

[B11] Elsevier (2022b). Journals Article Sharing – Policies. Elsevier. Available online at: https://www.elsevier.com/about/policies/sharing

[B12] EMARA (2022). Preprint Policy - EMARA: Indonesian Journal of Architecture. 10.29080/emara

[B13] Ettinger C. Sadanandappa M. K. Coghlan K. Hallenbeck K. K. Puebla I. (2022). A *Guide to Preprinting for Early Career Researchers*. OSF Preprints. 10.31219/osf.io/e59tk PMC9346271 35876380

[B14] Irawan D. E. Abraham J. (2021). Set them free. Commonplace. 10.21428/6ffd8432.5ee70f55

[B15] JAKI (2022). Self-Archieving - Indonesian Journal of Health Administration (Jurnal Administrasi Kesehatan Indonesia). Available online at: https://e-journal.unair.ac.id/JAKI/SLA

[B16] Kaiser J. (2022). NIH Enables Investigators to Include Draft Preprints in Grant Proposals. Available online at: https://www.science.org/content/article/nih-enables-investigators-include-draft-preprints-grant-proposals

[B17] Kurniawan P. S. (2019). Designing the initial indicators to implement the concept of sustainability accounting in small and medium enterprises: a conceptual approach. INA-Rxiv. 10.31227/osf.io/z4hsk

[B18] Kurniawan P. S. (2022a). Putu Sukma Kurniawan Youtube Channel. YouTube. Available online at: https://www.youtube.com/user/mwmw0000/featured

[B19] Kurniawan P. S. (2022b). The Conversation Author Profile - Putu Sukma Kurniawan. Conversation. Available online at: https://theconversation.com/profiles/putu-sukma-kurniawan-1035813

[B20] Mallapaty S. (2020). Popular preprint servers face closure because of money troubles. Nature 578:349. 10.1038/d41586-020-00363-3 32071446

[B21] Matthews D. (2018). New Boost for Preprints After Acceptance by ERC. Times Higher Education (THE). Available online at: https://www.timeshighereducation.com/news/new-boost-preprints-after-acceptance-erc

[B22] MDPI (2016). Introducing Preprints: A Multidisciplinary Open Access Preprint Platform. MDPI Blog. Available online at: https://blog.mdpi.com/2016/07/28/introducing-preprints-a-multidisciplinary-open-access-preprint-platform

[B23] Nosek B. (2022). Public Goods Infrastructure for Preprints and Innovation in Scholarly Communication. Available online at: https://www.cos.io/blog/public-goods-infrastructure-preprints-and-innovation-scholarly-communication

[B24] Onie S. (2020). Redesign open science for Asia, Africa and Latin America. Nature 587, 35–37. 10.1038/d41586-020-03052-3 33144703

[B25] Peer Community In (2022). Peer Community In - Free Peer Review & Validation of Preprints of Articles. Peer Community In. Available online at: https://peercommunityin.org

[B26] Research Square, (2022). Home. Available online at: https://www.researchsquare.com

[B27] Robson S. G. Baum M. A. Beaudry J. L. Beitner J. Brohmer H. Chin J. M.. (2021). Promoting open science: a holistic approach to changing behaviour. Collabra: Psychol. 7:e30137. 10.1525/collabra.30137

[B28] Scholarly Kitchen (2020). The Second Wave of Preprint Servers: How Can Publishers Keep Afloat? Scholarly Kitchen. Available online at: https://scholarlykitchen.sspnet.org/2019/10/16/the-second-wave-of-preprint-servers-how-can-publishers-keep-afloat

[B29] Science C for O (2022). Six New Preprint Services Join a Growing Community Across Disciplines to Accelerate Scholarly Communication. Available online at: https://www.cos.io/about/news/six-new-preprint-services-join-growing-community-across-disciplines-accelerate-scholarly-communication

[B30] Sever R. Roeder T. Hindle S. Sussman L. Black K.-J. Argentine J.. (2019). bioRxiv: the preprint server for biology. bioRxiv.

[B31] Singh-Shepherd S. (2022). Preprints and Proceedings B – the Story so Far - Royal Society. Available online at: https://royalsociety.org/blog/2021/07/preprints-and-proceedings-b-the-story-so-far

[B32] Springer S. (2022). Editorial policies - Preprint Sharing - Springer. Springer. Available online at: https://www.springer.com/gp/editorial-policies/preprint-sharing

[B33] Stagars M. (2016). Open Data in Southeast Asia. Cham: Springer International Publishing.

[B34] Synbio S. (2022). Welcome to Synbio.id. Available online at: https://synbio.id

[B35] UNESCO (2021). UNESCO Recommendation on Open Science. UNESCO. Available online at:https://unesdoc.unesco.org/ark:/48223/pf0000379949

[B36] UNESCO (2022). Letting the Sun Shine In: Transparency and Accountability in the Digital Age. UNESCO. Available online at: https://unesdoc.unesco.org/ark:/48223/pf0000377231

[B37] Wiley W. (2022). Preprints Policy. Wiley. Available online at: https://authorservices.wiley.com/author-resources/Journal-Authors/open-access/preprints-policy.html

